# The Difficult Clinical Decision of Thrombolytic Therapy for Submassive Pulmonary Embolism in a Community Hospital

**DOI:** 10.7759/cureus.11148

**Published:** 2020-10-25

**Authors:** Qian Zhang, Jonathan Vayalumkal, John Ricely, Daniel L Gray, Ahmad Raza

**Affiliations:** 1 Internal Medicine, Abington Hospital–Jefferson Health, Abington, USA

**Keywords:** systemic thrombolysis, submassive pulmonary embolism, acute pulmonary embolism

## Abstract

Submassive or intermediate-risk pulmonary embolism (PE) occurs when an acute PE episode is associated with radiographic evidence of right heart strain without hemodynamic instability. Further risk stratification is important in determining whether systemic thrombolytic therapy should be administered when weighing the risks and benefits. It includes the risk of death from acute PE versus the risk of bleeding. This decision could be further complicated in institutions where there is a lack of complete therapeutic options, which increases the importance of the expertise of a pulmonologist or an intensivist to decide whether rescue reperfusion is needed. We describe the case of a 34-year-old female patient with a history of right thigh abscess and diabetes mellitus who was admitted for diabetic ketoacidosis (DKA) along right thigh abscess status post-incision and drainage. She had a syncopal episode and was found to have submassive PE with right heart strain with stable hemodynamics and oxygen requirement. She tolerated systemic thrombolytic therapy without complications with a drastic improvement in her cardiac function post-treatment.

## Introduction

Pulmonary embolism (PE) occurs when the pulmonary artery or one or more of its associated branches are obstructed, leading to a decrease in perfusion necessary for adequate tissue oxygenation [1]. A high mortality and morbidity rate is associated with PE due to hemodynamic instability [2]. There are approximately 60,000-100,000 annual deaths in the United States due to PE, while 25% of the patient population experiences sudden death as the first presenting symptom [3]. The nomenclature of acute PE is associated with the patient's clinical status. Massive PE occurs when there is concurrent hemodynamic instability. Submassive PE or intermediate-risk PE occurs when hemodynamic instability is absent but if right ventricular heart strain is found on imaging. Lastly, low-risk PE presents when there is neither hemodynamic instability nor right ventricular heart strain [1]. Systemic thrombolytic therapy is one of the methods utilized to treat patients with massive or submassive PE.

## Case presentation

A 34-year-old woman with a past medical history of hypertension and insulin-dependent diabetes mellitus was admitted to the hospital with the chief complaint of worsening pain and swelling to her right upper/inner thigh for the past four days and associated nausea. She had a history of abscesses in this region that had previously responded to warm compresses. She was fully functional at baseline, denied smoking history, prolonged periods of immobilization, or the use of estrogen-containing products. She also denied any family history of hypercoagulability or known malignancy. On presentation, she was also complaining of polydipsia and polyuria. She had been checking her blood sugars at home for the past few days prior to the presentation, with measurements as high as 400 mg/dL (normal range: 70-130 mg/dL). She was afebrile and hemodynamically stable, with a blood pressure of 109/67 mmHg, heart rate of 90 beats per minute, respiratory rate of 17 breaths per minute, and oxygen saturation of 99% on room air (RA). Physical exam revealed an obese woman with a body mass index of 41.5 kg/m^2^ in no acute distress and with normal bilateral air entry without wheezing or crackles appreciated. She had a regular rate and rhythm without any murmur, rubs, and gallop noted. The patient did not appear to be volume overloaded as there were no findings of third heart sound (S3), jugular venous distention (JVD), or pitting edema.

**Figure 1:**
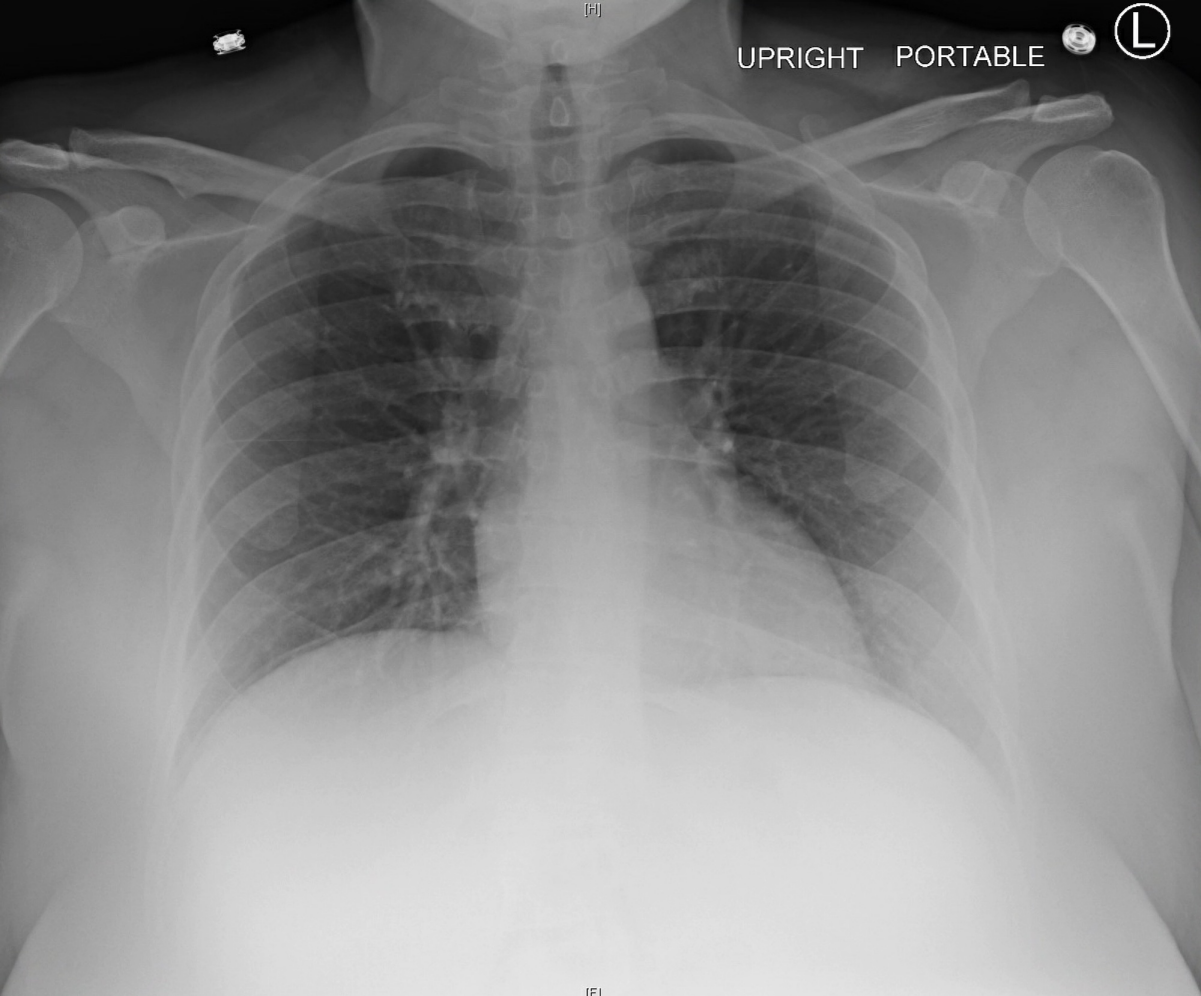
Chest X-ray of the patient No acute pulmonary disease noted L: left; [H]: head; [F]: foot

She had a wound with packing placed on her right inner thigh. The patient had undergone incision and drainage of the right thigh abscess in the emergency department. Serum creatinine was elevated to 1.30 mg/dL from the patient’s baseline of 1.01 mg/dL (normal range: 0.84-1.21 mg/dL). Glucose level was 519 mg/dL (normal range: 70-130 mg/dL), serum anion gap was 20 mEq/L (normal range: 7-13 mEq/L), and beta-hydroxybutyrate level was 64 mmol/L (normal range: <0.4 mmol/L). Beta human chorionic gonadotropin (HCG) urine test was unremarkable. Arterial blood gas (ABG) revealed metabolic acidosis with pH of 7.188 (normal range: 7.35-7.45), a carbon dioxide level of 33.3 mmHg (normal range: 35-45 mmHg), oxygen level of 50.4 mmHg (normal range: 75-100 mmHg), and a bicarbonate level of 12.2 mmol/L (normal range: 22-26 mmol/L). Serum hemoglobin was 14.4 g/dL (normal range: female: 12.0-16.0 g/dL). A chest radiograph revealed no active pulmonary disease (Figure 1). The patient was admitted to the progressive care unit (PCU) for the treatment of diabetic ketoacidosis (DKA) by the DKA protocol and right upper thigh abscess via vancomycin.

On day two of hospitalization, the patient’s anion gap had closed and she was transitioned from the insulin drip to long-acting insulin and an insulin sliding scale. Serum creatinine came down to 0.77 mg/dL (normal range: 0.84-1.21 mg/dL). Moreover, she reported lightheadedness upon standing along with general fatigue. She was afebrile, although she was slightly tachycardic with a heart rate of 120-125 beats per minute and marginal blood pressure of 90/60 mmHg. Out of concern for dehydration, the patient was given 500 mL of lactated Ringer’s solution. A rapid response alert was activated for a syncopal event as the patient had fallen on to the floor of the bathroom. She denied any pain, nausea, lightheadedness, palpitations, or urinary incontinence. Vitals included a heart rate of 132 beats per minute, blood pressure of 124/72 mmHg, respiratory rate of 28 breaths per minute, and oxygen saturation of 95% on 2-L nasal cannula (NC). She was tachycardic while the rest of her examination was unremarkable.

**Figure 2:**
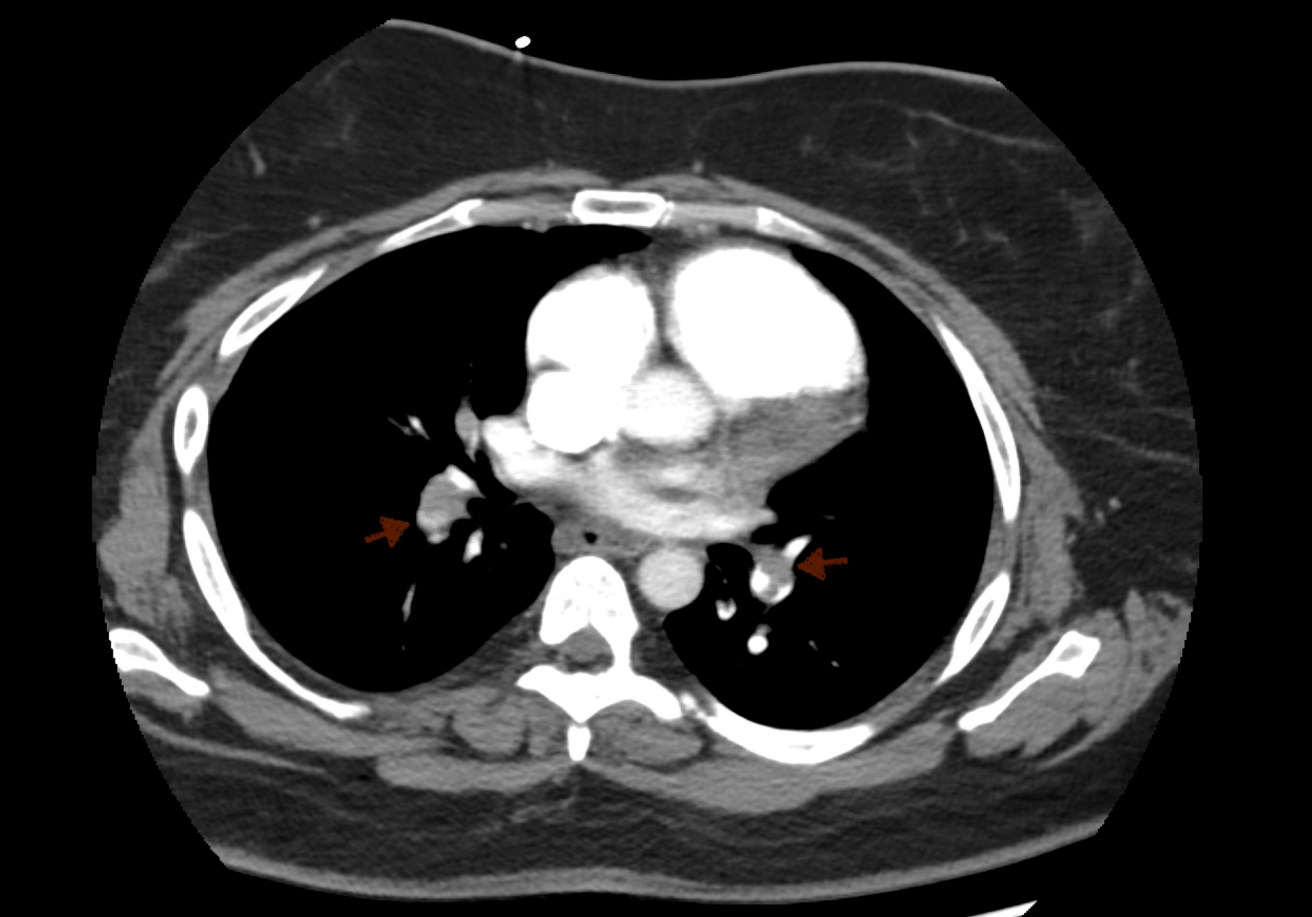
CT with pulmonary embolism protocol The image shows severe bilateral pulmonary embolism with associated right heart strain with compression of the left ventricle. Red arrows: pulmonary embolism CT: computed tomography

Labs revealed a leukocytosis of 12.6 K/uL (normal range: 4.0-11.0 K/μL), cardiac NT-proB-type natriuretic peptide (BNP) of 23 pg/mL (normal range: 0-99 pg/mL), and troponin I high sensitivity of 470 ng/L (normal range: <16 ng/L). CT scan of the head and CT thorax with PE protocol were ordered. Head CT was unremarkable. However, CT PE revealed severe bilateral PE with associated severe right heart strain with compression of the left ventricle (Figure 2). Lovenox was discontinued and the patient was started on a heparin drip while being upgraded to the medical intensive care unit (MICU) for the continuation of care. Initial transthoracic echocardiogram (TTE) revealed ejection fraction (EF) of 65-70% (normal range: >55%) with severe right ventricular dilation and interventricular septum flattening consistent with right ventricle overload. Right ventricular systolic function was also reduced, with right systolic ventricular pressure measured at 50 mmHg (normal range: 20-30 mmHg) and a dilated inferior vena cava also suggesting increased right atrial pressure. She continued to be hemodynamically stable while saturation was maintained on minimal oxygen supplementation. The decision to administer thrombolytic therapy was made given the patient's presentation, CT findings, and echocardiogram findings. The patient was given tissue plasminogen activator (tPA) 100 mg IV over two hours and then subsequently restarted on a heparin drip.

On day three of hospitalization, TTE compared to the one taken the previous day revealed decreased right ventricular size with significant improvement in right ventricular systolic function. The left ventricular function remained normal. The patient reported feeling much better, with no chest pain, shortness of breath, dizziness, or palpitations. She remained afebrile and hemodynamically stable while oxygen saturation of 100% was maintained on the 2-L NC. Serum creatinine was 0.70 mg/dL (normal range: 0.84-1.21 mg/dL) and serum hemoglobin was 10.9 g/dL (normal range: female: 12.0-16.0 g/dL). She was downgraded to the general medicine floors given her medically stable condition.

On days 4-10 of hospitalization, the patient was stable from a respiratory standpoint and was transitioned from NC to room air. She had minor bleeding at her right thigh incision and drainage site after having received thrombolytic therapy. The bleeding was later controlled without complications. Heparin drip was transitioned to oral anticoagulation. She was discharged home with close monitoring and follow-up appointments with the outpatient department.

She remained medically stable without complications at three months after hospital discharge. She denied chest pain, shortness of breath, palpitations, or noticeable bleeding. A complete review of systems was unremarkable. She is tolerating oral anticoagulants without any complications. She has stated that she is compliant with her follow-up appointments with various specialties.

## Discussion

Determining whether thrombolytic therapy should be administered or not in the setting of a submissive PE is an extremely difficult decision. The mortality from PE versus the risk of bleeding is the most important factor to be considered when weighing the risks and benefits. This challenging decision could be further complicated by the lack of resources at certain smaller community hospitals, especially when a referral center is far away or the patient is too unstable to tolerate transferring.

The PE response team (PERT) is composed of multidisciplinary specialties including, but not limited to, the pulmonologist, cardiologist, emergency medicine physicians, interventional radiologist, and cardiac surgeons; it responds appropriately in a timely fashion when a patient is diagnosed with massive or submassive PE. The team is designed to react in an organized manner in order to decrease PE-associated mortality and improve patient safety [4,5]. PERT differs greatly among different institutions when it comes to specific algorithms, resources, and effectiveness. However, this team is generally able to decrease the controversial elements regarding the treatment of patients with submassive PE. Our hospital, similar to many of the hospitals across the United States, lacks a PERT rapid response system, which increases the complexity of treating submassive PE. Pulmonologists and intensivists are often placed in a difficult position when their proper, timely clinical judgment could affect the patient's prognosis. Unfortunately, the treatment strategy could further be limited in institutions where catheter-directed therapy is unavailable as a means of treatment.

A detailed, individualized, and holistic approach must be initiated when first assessing a patient with radiographically confirmed PE. The first step is to assess the risk of death of the patient from PE. Patients are stratified into three different categories: high, intermediate, and low risk. The high-risk population, also known as massive PE, includes those with hemodynamic instability, refractory shock, or cardiac arrest. These patients are candidates for thrombolytic therapy if the risk of bleeding is low. Low-risk patients are often simply treated with anticoagulation without reperfusion therapy. However, patients who are categorized into the intermediate-risk group or submassive PE are the ones who often present the most challenging scenarios [6]. Submassive PE or intermediate-risk PE occurs when hemodynamic instability is absent but if right ventricular heart strain is found on imaging [1].

The PE severity index (PESI) is a commonly used prognostic formula by clinicians to predict the 30-day mortality in patients with PE. It was later translated into a simplified PESI (sPESI) version that allows rapid interpretation of the patient's clinical status. It factors in six objective markers with each correlating to a single point. It includes age of >80 years, history of chronic cardiopulmonary disease, history of cancer, heart rate of >110 beats/minute, systolic blood pressure of <100 mmHg, and arterial oxyhemoglobin saturation of <90%. Patients with 0 points are considered to be low-risk, while those with a score equal to or greater than 1 point is classified into the high-risk group [7,8]. 

Moreover, patients with submassive PE, the intermediate-risk category, are further divided into intermediate-low risk PE and intermediate-high risk PE subgroups. Patients with an abnormal right ventricular function on echocardiogram or CT chest in addition to an elevated cardiac BNP or troponin level are considered to be in the intermediate-high risk category. On the other hand, patients with either right ventricular function abnormality or elevated cardiac BNP or troponin levels are classified into the intermediate-low risk category. Patients in the intermediate-high risk subgroup are recommended to be managed with rescue reperfusion such as systemic thrombolysis or surgical embolectomy versus percutaneous catheter-directed therapy. Furthermore, patients in the intermediate-low risk subgroup are recommended to be treated with anticoagulation with close monitoring. Our patient had the presence of right ventricular dysfunction on echocardiogram as well as elevated troponin level, and hence was considered to receive systemic thrombolysis. In addition, our patient had an sPESI score of >1, which also put her in the intermediate-high risk group [6].

However, the decision on whether to administer systemic thrombolysis also depends largely on the patient's bleeding risk profile along with the presence of contraindications. Absolute contraindications to systemic thrombolysis include, but are not limited to, the presence of an intracranial neoplasm, intracranial or spinal surgery within the past two months, history of hemorrhagic stroke, severe thrombocytopenia, presence of active bleeding, or ischemic stroke in the past three months. Other relative contraindications are uncontrolled severe hypertension, pregnancy, or recent surgery within the previous 10 days [9]. Our patient had undergone a recent incision and drainage of her right thigh abscess a couple of days prior to the acute submissive PE episode despite not having other notable contraindications. Our hospital is currently not equipped with the catheter or surgical embolectomy treatment option, which increased the complexity of the decision on whether systemic thrombolysis should be administered. However, the risk stratification and the bleeding risk profile can only serve as supportive evidence for the clinician. It is more important to assess the patient holistically and individualize each treatment plan. Our patient tolerated the full dose of systemic thrombolytic therapy without significant bleeding except for minor bleeding at the right thigh incision site. Her repeat echocardiogram showed significant improvement in the right heart function. She was subsequently discharged without complications and remained to be asymptomatic three months after the hospitalization.

## Conclusions

Patients with submassive or intermediate-risk PE should be evaluated holistically. Moreover, formulating a treatment plan could be extremely complicated in hospitals without a PERT or with limitations in other therapeutic resources. The expertise of a pulmonologist or intensivist in treating submassive PE could be a game-changing factor in terms of the patient's outcome and prognosis. Every treatment plan should be individualized while using risk stratification and bleeding risk profile as valuable clinical supporting pieces of evidence.
